# MicroRNA-196a Is a Putative Diagnostic Biomarker and Therapeutic Target for Laryngeal Cancer

**DOI:** 10.1371/journal.pone.0071480

**Published:** 2013-08-14

**Authors:** Koichiro Saito, Koji Inagaki, Takahiro Kamimoto, Yoko Ito, Toshiaki Sugita, Satoko Nakajo, Akira Hirasawa, Arifumi Iwamaru, Takashi Ishikura, Hideki Hanaoka, Keisuke Okubo, Tokio Onozaki, Takeru Zama

**Affiliations:** 1 Department of Otolaryngology-Head and Neck Surgery, Keio University School of Medicine, Tokyo, Japan; 2 Genetic Testing Section, Center for Genetic & Chromosomal Analysis, SRL, Inc., Tokyo, Japan; 3 Department of Obstetrics and Gynecology, Keio University School of Medicine, Tokyo, Japan; 4 Department of Surgery, Federation of National Public Service Personnel Mutual Aid Associations Tachikawa Hospital, Tokyo, Japan; 5 Life Technologies Japan, Tokyo, Japan; 6 Department of Otolaryngology, Sano Kousei General Hospital, Tochigi, Japan; The George Washington University, United States of America

## Abstract

**Background:**

MicroRNA (miRNA) is an emerging subclass of small non-coding RNAs that regulates gene expression and has a pivotal role for many physiological processes including cancer development. Recent reports revealed the role of miRNAs as ideal biomarkers and therapeutic targets due to their tissue- or disease-specific nature. Head and neck cancer (HNC) is a major cause of cancer-related mortality and morbidity, and laryngeal cancer has the highest incidence in it. However, the molecular mechanisms involved in laryngeal cancer development remain to be known and highly sensitive biomarkers and novel promising therapy is necessary.

**Methodology/Principal Findings:**

To explore laryngeal cancer-specific miRNAs, RNA from 5 laryngeal surgical specimens including cancer and non-cancer tissues were hybridized to microarray carrying 723 human miRNAs. The resultant differentially expressed miRNAs were further tested by using quantitative real time PCR (qRT-PCR) on 43 laryngeal tissue samples including cancers, noncancerous counterparts, benign diseases and precancerous dysplasias. Significant expressional differences between matched pairs were reproduced in miR-133b, miR-455-5p, and miR-196a, among which miR-196a being the most promising cancer biomarker as validated by qRT-PCR analyses on additional 84 tissue samples. Deep sequencing analysis revealed both quantitative and qualitative deviation of miR-196a isomiR expression in laryngeal cancer. In situ hybridization confirmed laryngeal cancer-specific expression of miR-196a in both cancer and cancer stroma cells. Finally, inhibition of miR-196a counteracted cancer cell proliferation in both laryngeal cancer-derived cells and mouse xenograft model.

**Conclusions/Significance:**

Our study provided the possibilities that miR-196a might be very useful in diagnosing and treating laryngeal cancer.

## Introduction

In 2004, 28,260 new cases of oral cavity and pharyngeal cancer and 20,260 new cases of laryngeal cancer were diagnosed in the United States, resulting in 7,230 and 3,830 deaths, respectively [1], [2]. Despite significant advances in surgery and radiotherapy over the last decades, the 5-year survival rates of head and neck squamous cell carcinoma (HNSCC) patients have been improved only moderately in part due to the relatively high local recurrence rate. At present, locoregional HNC is treated with a combination of surgery and radiation with or without chemotherapy, while each treatment option results in devastating consequences on speech and swallowing function. In addition, surgical procedures in the head and neck and oral cavity region commonly result in significant cosmetic deformities. Even with the combined treatment approaches mentioned, patients with advanced HNC are thus in need of novel and less invasive treatments for their high morbidity disease. In this study, thinking about considerable heterogeneity of HNSCC tumors, we have focused on a single well-defined anatomical location, larynx. While laryngeal cancer is highly curable either by surgical removal or irradiation when found and treated at the early stage, advanced cancer stays much less curable resulting in no significant improvement of overall survival rates since 1975 [3]. Thus, highly sensitive biomarkers to detect laryngeal cancer even at the early stage without clinical symptoms, and significantly effective novel therapeutic agents are necessary to further improve patient outcomes of laryngeal cancer. Furthermore, current approaches to predict the outcome of HNSCC patients include examination using clinicopathological parameters such as primary tumor, regional node, distant metastasis (TNM) – stage, depth of invasion, and differentiation grade. However, these parameters do not accurately reflect prognosis of the patients and additional predictors and biomarkers would be useful for patient management. Thus, molecular and cellular biology is a promising field of study that may lead to the discovery of novel biomarkers and novel therapeutic targets.

MicroRNA (miRNA), which encodes a small non-coding RNA of ∼22 nucleotides, is now recognized as a large gene family expressed in plants, animals, and viruses as well as in unicellular algae [4]. Most animal miRNAs are evolutionarily conserved and often found in clusters [5]. Primary miRNAs (pri-miRNAs) forming stem-loop structures are predominantly transcribed by RNA polymerase II and are successively processed by two RNase III-like enzymes, Drosha in the cell nucleus and Dicer in the cell cytoplasm, to generate mature miRNAs [6], [7]. miRNAs negatively regulate gene expression at the posttranscriptional level by cleavage and/or translational repression of their mRNA targets via interaction using perfect base pairing in 5′ end of the mature miRNA, also termed the seed region [8]. Recent bioinformatics analyses reported that over 60% of protein-coding genes have potential to pair with and to be controlled by miRNAs [9]. Further investigations have demonstrated that miRNAs play extremely important roles in almost all aspects of biology, including metabolism, cell proliferation, apoptosis, development and differentiation [10], [11].

In recent years, there has been a considerable interest in understanding the role of miRNAs in disease processes and their dysregulation is believed to promote the malignant behavior of tumors [12]. The links between the aberrant expression of miRNAs and the pathogenesis of several cancer types[12]–[14] are documented.

It has also been reported that miRNAs could be an ideal biomarkers for cancer detection because: (i) miRNA expression is frequently dysregulated in cancer [15], [16], (ii) expression patterns of miRNAs in human cancer appear to be tissue-specific [17], and (iii) miRNAs have unusually high stability even in formalin-fixed tissues [18]. Furthermore, recent extensive miRNA research has also revealed that miRNAs are promising therapeutic targets in cancers including colon, breast, gastric and hepatocellular malignancies[19]–[26].

All of these issues are important for providing a deeper understanding of the roles of miRNAs in HNSCC pathogenesis and as potential prognostic and diagnostic markers as well as therapeutic targets in HNSCC. In this study, we have profiled the expression of 723 unique human miRNAs in 3 laryngeal cancers, 2 noncancerous laryngeal counterparts using oligonucleotide microarrays with a hairpin structure incorporated onto the 5′ end of the probe [27] fabricated by an ink-jet oligonucleotide synthesizer [28] (Agilent Human miRNA V2, Agilent Technologies) and identified several miRNAs that could serve as potential diagnostic disease markers. We further confirmed the expression of selected miRNAs using qRT-PCR (TaqMan® miRNA assays, Applied Biosystems) in 5 noncancerous counterparts, 14 polyps or nodules, 12 dysplasias and 17 laryngeal cancers to provide data suggesting that altered expression levels of miRNAs have a functional consequence in laryngeal cancer. *In situ* hybridization (ISH) further proved the markedly differential expression of miRNAs in cancer lesion compared to normal squamous cell epithelium.

We further assessed the impact of miR-196a inhibition *in* *vitro* and *in* *vivo* on the growth of HNSCC to evaluate the potential of miRNA as a novel therapeutic target for HNC.

## Materials and Methods

### Ethics Statement

The research protocol of this study was approved by the institutional review board of the Keio University School of Medicine and Sano Kousei General Hospital. All tissue samples were taken for the purpose of diagnostic biopsy or treatments from the patients who underwent surgery after approval of the study by the institutional review board at the Keio University School of Medicine and Sano Kousei General Hospital. Written informed consent was obtained from all patients.

Animals were cared for and used in accordance with protocols approved by the Animal Care and Use Committee of Keio University School of Medicine (Permit number for this study: 10243– (0)). All surgery was performed under tribromoethanol anesthesia, and all efforts were made to minimize suffering.

### Patients and Specimens

A part of the sample was processed for formalin-fixed and paraffin-embedded (FFPE) procedure, followed by routine pathologic analyses, while the residual tissue was saturated in RNA*later*® (Ambion, Foster City, CA) and stored at −80°C until processing. All FFPE samples were reviewed by experienced pathologists to confirm the diagnoses. Patient characteristics and pathologic staging are summarized in Table 1.

**Table 1:** List of analyzed tissue samples.

Sample		ID
**Noncancerous counterpart**		*T469* *, *T479* *, T529*, T539*, T542*, **T563** *, **T591** *, **T596** *, **T613** *, **T621** *, **T634** *, **T656**, **T709** *, **T790** *, **T792**
**Benign**	**Polyp and Nodule**	T436, T440, T458, T462, T480, T522, T534, T568, T569, T577, T578, **T647**
	**Polypoid**	T438, T446
	**Hyperkeratosis**	T543, **T793**
	**Papilloma**	**T483**, **T497**, **T499**, **T501**, **T615**, **T649**, **T665**, **T711**
**Dysplasia**	**Mild**	T413, T431, T455, T481, T532, T540, **T672**
	**Moderate**	T482, **T585**, **T635**, **T679**, **T815**
	**Severe**	T427, T444, T498, T515, T551, **T760**
**Cancer**	**T1a**	T541*, T544, **T612** *, **T620** *, **T789** *
	**T1b**	T433, *T478* *, **T592** *, **T633** *, **T800**
	**T2**	T423, *T468* *, T491, T528*, T536*, T547, **T595** *, **T708** *
	**T3**	T415, T505 (N2c), T524 (N1), T527 (N1), **T603 (N2c)**
	**T4**	T407 (N2b), T517 (N2c), **T560 (N2b)** *
	**rT1b**	*T485*
	**HNSCC cell lines**	HSC-2 (oral), HSC-3 (oral), HSC-4 (oral), SAS (tongue), Ca9-22 (gingiva), JHU-011 (larynx), FaDu (hypopharynx)

**Figure 1:**
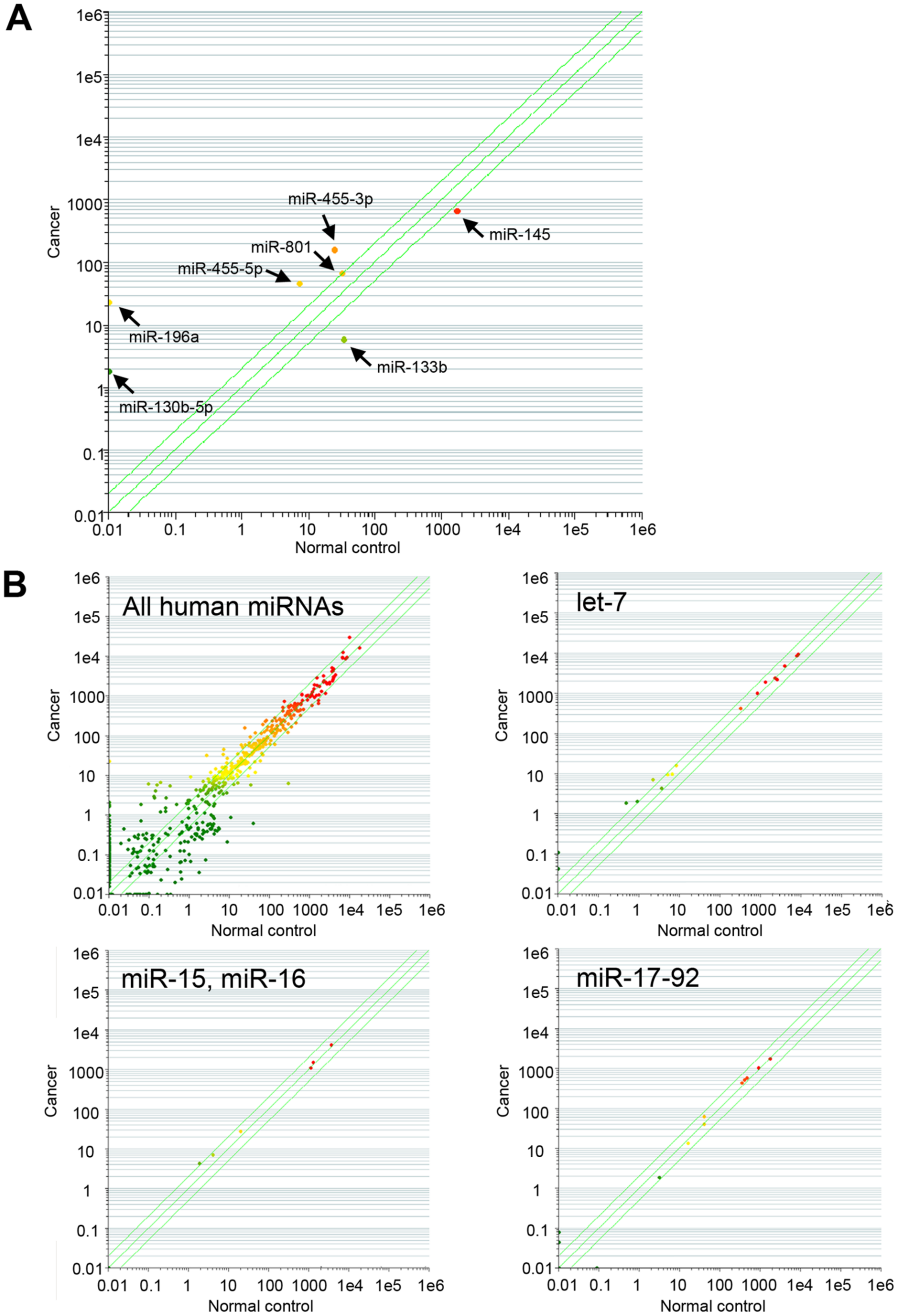
Microarray-based screen for miRNAs up- or down-regulated in laryngeal cancer. Datasets are compared between cancers and adjacent noncancerous counterparts, plotting the abundance of a given miRNA in one dataset against its corresponding value in another dataset both on a log scale. 723 human miRNAs were included in the Agilent microarray. (A) Comparison between normal controls and cancer samples demonstrated the up-regulation of 5 miRNAs (miR-130b-5p, miR-196a, miR-455-3p, miR-455-5p, and miR-801) and the down-regulation of 2 miRNAs (miR-133b and miR-145). (B) Expression levels of let-7 family, miR-15, miR-16, or miR-17-92 cluster, known to be involved in various other cancers, were not significantly different between normal controls and cancers (*upper right panel* and *lower panels*). Expression levels of 723 human miRNAs are also shown (*upper left panel*).

A total of 84 clinical tissues were included in the present study, of which 5 (*italics*) were screened by using the Agilent Human miRNA Microarray Kit V2 as shown in Figure 1, and 36 (*bold*) were subjected to qRT-PCR analysis for miR-196a quantification as shown in Figure 4. Seven HNSCC cancer cell lines were also subjected to miRNA qRT-PCR analysis. r, recurrent tumor;

* paired cancer samples and neighboring normal controls from 13 laryngeal cancer patients.

### Reagents

Unless otherwise noted, routine chemical materials were obtained from either Sigma-Aldrich (Saint Louis, MO) or Wako (Osaka, Japan). miR-196a inhibitor (hsa-miR-196a miRIDIAN Hairpin Inhibitor, IH-300529-06) and inhibitor negative control (control, miRIDIAN microRNA Inhibitor Negative Control no. 1, IN-001005-01) used in this study were purchased from Dharmacon (Lafayette, CO). miRIDIAN Hairpin Inhibitor was designed to inhibit the RISC function of the target miRNA with its double-stranded region [29]. For *in situ* hybridization (ISH) of miR-196a, the 3′-DIG-labeled locked nucleic acid-incorporated miRNA probe (miRCURY LNA™ microRNA Detection Probe, Exiqon, Vedbaek, Denmark) was used in this study.

### RNA Extraction and Microarray Analysis of miRNA

Total RNA including low molecular weight RNA from tissue samples and cancer cell lines was isolated using the mirVana™ miRNA Isolation Kit (Ambion) according to the manufacturer’s instructions. The quality of the RNA samples was assessed using an Agilent 2100 Bioanalyzer, and only the samples meeting the criteria of 28S/18S >1 and RNA Integrity Number (RIN) ≥7.5 were used for all analyses.

For microarray analysis, we used the Human miRNA Microarray Kit V2 (Agilent Technologies, Santa Clara, CA), which contains 20–40 features targeting each of 723 human miRNAs (Agilent design ID 019118) [30] as annotated in the Sanger miRBase, release 10.1 [31]. Labeling and hybridization of total RNA samples were performed according to the manufacturer’s protocol. One hundred ng of total RNA was used as an input into the labeling reaction, and the entire reaction was hybridized to each array for 20 hours at 55°C. The results were analyzed using Agilent GeneSpring GX7.3. Normal controls and cancer samples were compared using Welch’s t-test (p<0.05) and differentially expressed miRNAs with at least a 2-fold change in expression were considered to be potential biomarkers. All in-house data of microarray analysis of miRNAs were deposited in the Gene Expression Omnibus (GEO) with an accession number of GSE47610.

### TaqMan® Quantitative Real-Time PCR Assay of miRNAs

The expression levels of each miRNA were confirmed with TaqMan® Quantitative Real-Time PCR (qRT-PCR) Assay using individual specific primers and probes according to manufacturer’s instructions (Applied Biosystems, Foster City, CA). Briefly, 10 ng of total RNA was reverse transcribed with miRNA-specific stem-loop primers (Applied Biosystems), and the PCR was executed with the generated RNA-specific cDNA in gene-specific TaqMan® miRNA real-time PCR assay solution on a StepOnePlus Real-Time PCR System (Applied Biosystems). The reaction was performed at 95°C for 10 min, followed by 45 cycles of 95°C for 15 s, and 60°C for 60 s. All samples were measured in triplicate, including no template controls. Normalization was performed with the small nuclear RNA U6 (RNU6B; Applied Biosystems), and relative expression was calculated using the comparative Ct (Cycle threshold) method. Statistical analyses were performed using paired t-test to compare the expression levels in matched pairs and Tukey-Kramer test to compare the expression levels between multiple diseases and further analyses were performed by pairwise Mann-Whitney’s U test. The significance level was set at p<0.05.

### Next Generation Sequencing of miRNAs

Next generation sequencing analysis was carried out by using Applied Biosystems SOLiD™ system and SOLiD™ Small RNA Expression Kit according to SOLiD™ System 3 User Guide (Applied Biosystems). The percentage of small RNA per total RNA mass was calculated by Agilent 2100 Bioanalyzer. The small RNA content was enriched using flashPAGE™ fractionation (Ambion) if needed. The small RNA species (10–40 nt) were then converted to cDNA libraries using SOLiD™ Small RNA Expression Kit (Applied Biosystems), followed by cleanup and size selection by PAGE around ∼105–150 bp. Clonal amplification onto beads with emulsion PCR was performed using the SOLiD™ ePCR kit. Prepared beads were subjected to quality check run and were then sequenced on SOLiD™ 3 Analyzer. Total of 11.6 to 35.9 million sequence reads were obtained from each library, and downstream analysis was carried out by using pipeline SOLiD™ System small RNA Analysis Pipeline Tool (corona RNA2MAP version 0.50). All reads were mapped sequentially against (1) ribosomal RNA and transfer RNA, (2) known reference sequences of miRNAs in Sanger miRBase Release 18, (3) hg19 human reference genome assembly (Genome Reference Consortium GRCh37) as well as human mitochondrial genome (NC_001807.4). The sum of the number of short reads that mapped to each region of interest was normalized to the total reference-mapped short reads in each library sample, and used as raw expression values of the corresponding region. Conservation and repeat information was obtained by using the University of California Santa Cruz table browser. Samples analyzed include 2 laryngeal cancers (T416, 66 y.o. male, T3N0; T506, 74 y.o. male, T3N2c) as advanced cancer samples and 3 laryngeal papillomas (T421, 33 y.o. male, adult onset; T484, 33 y.o. male, adult onset; T502, 38 y.o. male, adult onset) as non-cancer samples. All in-house data of next generation sequencing of miRNAs were deposited in the DDBJ Sequence Read Archive (SRA) with an accession number of PRJDB994.

### *In situ* Hybridization for miR-196a

The *in situ* detection was performed on serial 4-µm paraffin sections including normal mucosa and laryngeal cancer material of a 74 y.o. male patient who underwent total laryngectomy for complete removal of the advanced T3 tumor. Sections were deparaffinized and subsequently digested with proteinase K (10 µg/ml) for 5 min at 37°C. After proteinase K digestion, the sections were fixed in 4% paraformaldehyde, and the slides were then hybridized with 4 pmol Exiqon’s miRCURY LNA detection probe complementary to miR-196a in hybridization buffer (50% formamide, 5×SSC, 0.1% Tween, 9.2 mM citric acid (pH 6), 50 µg/ml heparin, and 500 µg/ml yeast RNA) overnight at 50°C. A scrambled and an RNU6B probe (Exiqon) were also used as negative and positive controls, respectively. Then, after incubation with anti–DIG-AP Fab fragments conjugated to alkaline phosphatase diluted 1∶50 in blocking solution (Roche, Basel, Switzerland) for 3 hours at room temperature, the hybridized probes were detected by applying nitroblue tetrazolium chloride/5-bromo-4-chloro-3-indolyl phosphate color substrate (Roche) to the slides. Slides were counterstained with nuclear fast red and analyzed using a Nikon ECLIPSE 55i microscope.

### Cell Culture and Transfection

Head and neck squamous cell carcinoma (HNSCC) cell lines, derived from tongue (HSC-2, HSC-3, HSC-4), gingiva (SAS), the oral cavity (Ca9-22), larynx (JHU-011) and pharynx (FaDu), were all maintained in RPMI-1640 medium supplemented with 10% heat-inactivated fetal bovine serum and 1% penicillin-streptomycin at 37°C in 5% CO_2_.

For transfection assays, cells were transfected with either miR-196a inhibitor or inhibitor negative control using Lipofectamine™ 2000 reagent (Invitrogen, Carlsbad, CA) according to the manufacturer’s instructions. The inhibitor negative control was based on C. elegans miRNA and corresponds to cel-miR-239b. The control has been analyzed by BLAST against all human, mouse and rat genomic sequences and miRNA sequences in the miRBase sequence database. This molecule has identical design and modifications as miRIDIAN microRNA inhibitors that are known to target mRNA. Each experiment was performed in triplicate and independently at least 3 times.

### Cell Viability and Cytotoxicity Assay

JHU-011 cells were plated in a 24-well plate at a density of 2×10^4^ cells/well and seeded for 24 hours. Then, the cells were transfected as described above and cell proliferation was determined by direct cell counting at 0, 3 and 5 days after transfection. Briefly, cells were trypsinized and stained with 0.4% trypan blue to measure the extent of cell death using Countess™ (Invitrogen). A nuclear counterstaining using Hoechst 33258 was also performed to visualize the viable cells at 3 days after transfection.

Furthermore, cell viability and cytotoxicity were assessed by differential protease activities using MultiTox-Fluor Multiplex Cytotoxicity Assay (Promega, Fitchburg, WI) according to the manufacturer’s instructions. Briefly, the cells were seeded at the concentration of 5×10^3^ cells/well in a 96 well plate and transfection procedures were performed 24 hours after seeding. Then, the live cell protease activity and dead cell protease activity were measured at 3 days after transfection by adding glycyl-phenylalanyl-aminofluorocoumarin (GF-AFC) or bis-alanyl-alanyl-pnenylalanyl-rhodamine 110 (bis-AAF-R110) as peptide substrates, respectively. Cells were incubated for 30 minutes at 37°C and resulting fluorescence was measured (live-cell fluorescence 400_Ex_/505_Em_; dead-cell fluorescence 485_Ex_/520_Em_) to obtain the Relative Fluorescence Units (RFU).

### Laryngeal Cancer Xenograft Models

Experiments were performed on 6-week-old male BALB/c nude mice nu/nu. Animals were cared for and used in accordance with protocols approved by the Animal Care and Use Committee of Keio University School of Medicine (Tokyo, Japan). Ten mice were randomly divided into 2 groups of 5 mice. Efficacy of treatment on both tumors and their locoregional lymph node metastasis was examined in each of the following 2 groups: Group 1, miR-196a inhibitor; Group 2, inhibitor negative control (control). Mice were anesthetized by intraperitoneal injection of 5–7 mg tribromoethanol. Orthotopic xenograft tumors were established around the neck at the level of larynx by subcutaneous injection of 5×10^6^ JHU-011 cells with a 25 gauge needle. After one week, tumors were measured in three dimensions using calipers, and then either miR-196a inhibitor or control combined with AteloGene™ (KOKEN, Tokyo, Japan) (1 nmol/200 µl) was injected into the subcutaneous spaces around the tumors. Additional treatments were performed on 13 and 19 days after inoculation of tumor cells and periodical measurement of the tumor was performed up to 12 weeks after injection. Mice were euthanized and tumor masses were harvested, then cut into 2 pieces. Half of each tumor was fixed in 10% neutral buffered formalin overnight, dehydrated with step-by-step ethanol, and embedded in paraffin. Residual half of the tumor was saturated in RNA*later*® (Ambion) and stored at −80°C. Bilateral cervical lymph nodes were harvested, snap-frozen and paraffin embedded. Hematoxylin-Eosin staining was performed on paraffin embedded samples for histological analyses. Statistical analyses were performed using t-test to compare the therapeutic effect of miR-196a inhibitor against laryngeal cancer *in* *vitro* and *in* *vivo*. The significance level was set at p<0.05.

## Results

### Microarray Screening of Altered miRNA Expression in Laryngeal Cancer

To identify miRNAs dysregulated in laryngeal cancers, we first examined the expression profiles of 723 human mature miRNAs in 5 laryngeal tissues (3 laryngeal cancers and 2 adjacent noncancerous laryngeal counterparts) using microarrays. The results showed that 5 miRNAs (miR-130b-5p (formerly designated as miR-130b*), miR-196a, miR-455-3p, miR-455-5p, and miR-801) or 2 miRNAs (miR-133b and miR-145) were significantly up-regulated or down-regulated, respectively in laryngeal cancers (Figure 1A). Interestingly, expression levels of either let-7 family, miR-15, miR-16, or miR-17-92 cluster, known to be associated with various other cancers, were not obviously different between laryngeal cancer tissues and other noncancerous tissues (Figure 1B).

### Quantitative Real-time PCR Validation of miRNA Expression in Laryngeal Cancer

Next, to confirm and validate the findings obtained from our microarray analysis, we performed quantitative real-time PCR (qRT-PCR) analysis (TaqMan® MicroRNA Assays, Applied Biosystems) using 5 pairs of primary laryngeal cancers and their matched noncancerous tissues. Although microarray analysis revealed up-regulation of 5 miRNAs and down-regulation of 2 miRNAs, miR-801 is now considered to be a fragment of U11 spliceosomal RNA and removed from the miRBase [31]. miR-145 was also excluded from the further studies, because it has been reported to be frequently down-regulated in cancers in multiple organs including prostate cancer [32], breast cancer [33], bladder cancer [34], colon cancer [35], [36], ovarian cancer [37], esophageal cancer [38], lung cancer [39], [40], nasopharyngeal cancer [41], gastric cancer [42], as well as B-cell malignancies [43] and considered to be not a proper biomarker for laryngeal cancer.

**Figure 2:**
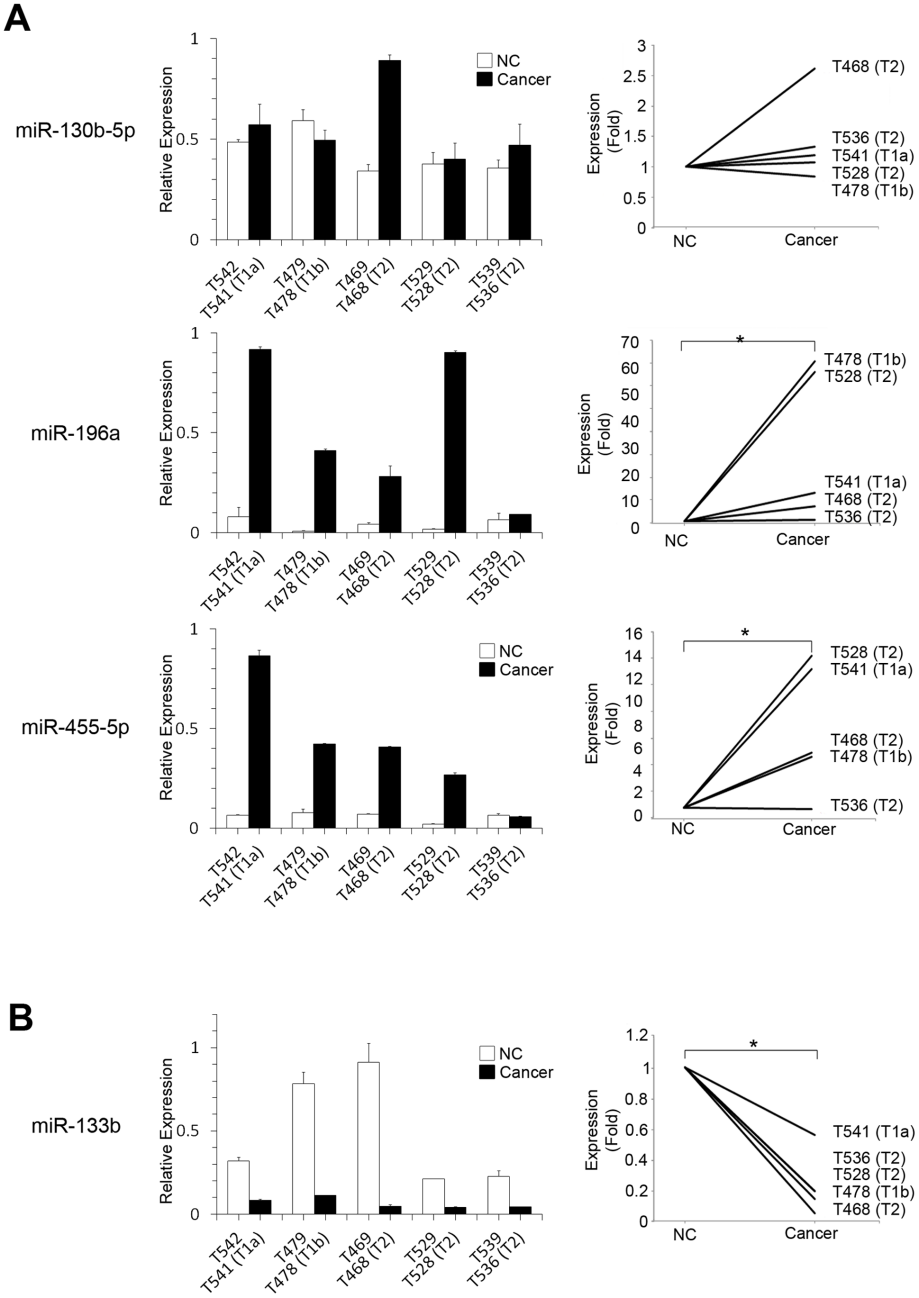
Differential expression of laryngeal cancer-associated miRNAs between matched paired samples. Relative expressions of laryngeal cancer-associated miRNAs in 5 paired samples were measured by TaqMan® qRT-PCR analysis and shown in *left panels*. Expression levels in adjacent noncancerous counterparts (NCs) in each pair are set to be 1 in *right panels* for clear visualization of significant fold difference in each miRNA. Data are expressed as mean values with standard deviations. (A) Up-regulation of 2 miRNAs (miR-196a and miR-455-5p) was confirmed in cancers when 5 cancers were compared with their NCs. *, *p*<0.05. (B) miR-133b was down-regulated in cancers in all 5 matched pairs. Statistically significant differences of the expression levels of miR-196a, miR-455-5p, and miR-133b were observed between matched pairs. *, *p*<0.05.

miR-455-5p instead of miR-455-3p was used in further studies, because miRNAs 3p and 5p are generated from either side of the same precursor stem to have similar sequences and recent report suggested the potential involvement of miR-455-5p in cancer progression [44]. Thus, qRT-PCR analysis was performed on residual 4 miRNAs (i.e., miR-130b-5p, miR-196a, miR-455-5p, and miR-133b). Expression levels of these miRNAs were compared between 5 cancer tissues and their adjacent noncancerous tissues. The results were consistent with those obtained from microarray analyses, especially when matched samples were compared in the same patients (Figures 2A and B). Higher expression levels of miR-130b-5p were observed in cancer tissues compared with neighboring controls in 4 of 5 pairs. Furthermore, expression levels of miR-196a and miR-455-5p were significantly higher in cancer tissues when compared with neighboring controls (miR-196a, p = 0.0460; miR-455-5p, p = 0.0286) (Figure 2A), while expression level of miR-133b was significantly lower in cancer samples compared with controls (p = 0.0274) (Figure 2B).

**Figure 3:**
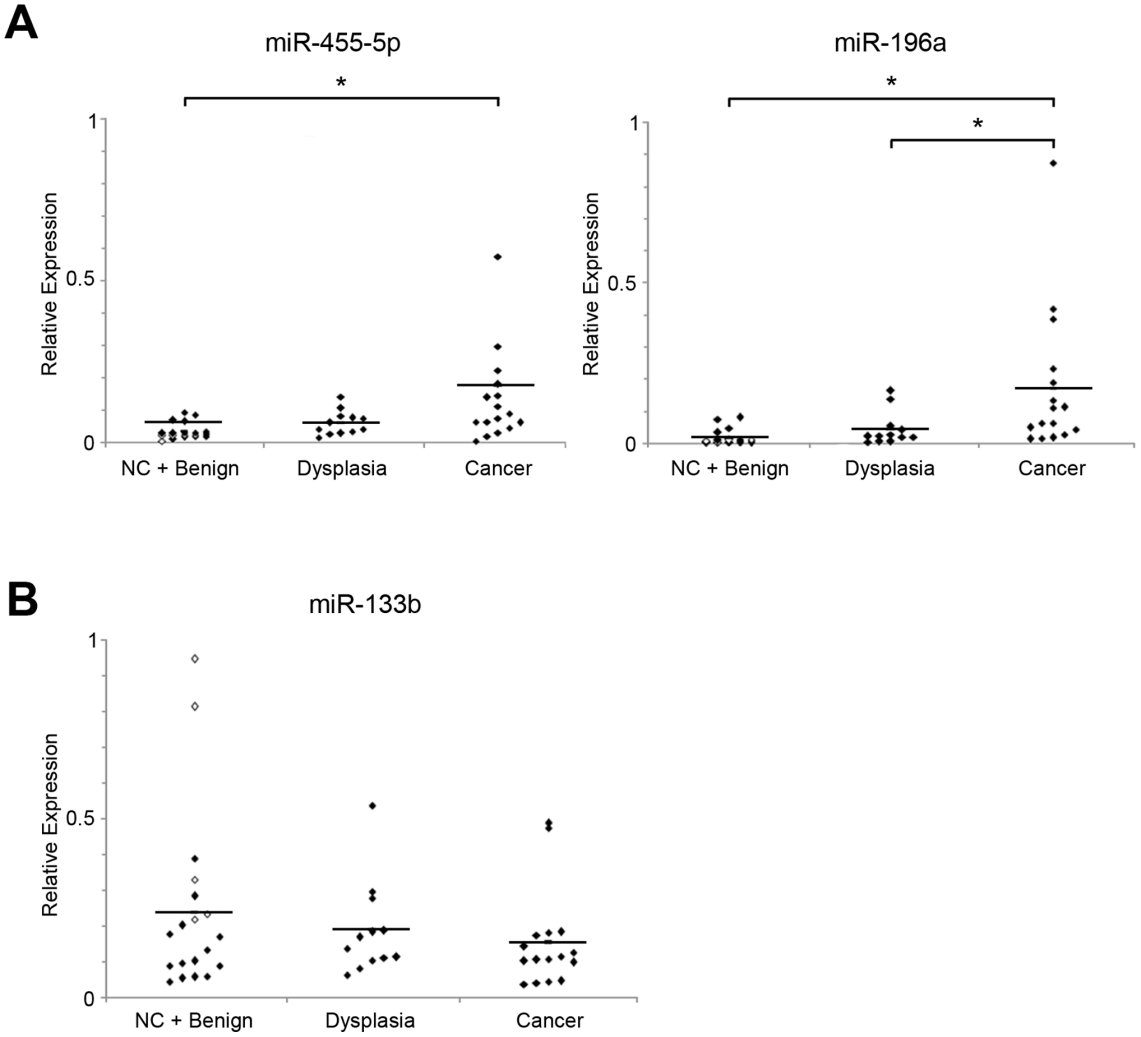
Significant up-regulation of miR-455-5p and miR-196a in multiple laryngeal cancer samples. (A) Expression levels of miR-455-5p and miR-196a were measured by TaqMan® qRT-PCR using 48 tissue samples. Adjacent noncancerous tissues (noncancerous counterparts, NCs) are shown as open quadrangles. Significant up-regulation of miR-455-5p was observed in cancers compared with NCs and benign samples. Expression levels of miR-196a were significantly higher in cancer tissues when compared with either NCs and benign samples or precancerous dysplasia samples. Bars indicate mean value of each group. *, *p*<0.05. (B) Expression levels of miR-133b were also examined in multiple samples. Although relatively lower expression was observed in cancers, the differences were not statistically significant when compared with other groups. Bars indicate mean value of each group. *, *p*<0.05.

Thus, of these 4 miRNAs, 3 miRNAs (i.e., miR-196a, miR-455-5p and miR-133b) showed significantly different expression levels in cancer tissues when compared with their matched control tissues and further quantification of miRNAs was performed using 48 laryngeal samples. These specimens included 5 noncancerous counterparts, 14 benign lesions (polyp, nodule, polypoid, or hyperkeratosis), 12 dysplasias, and 17 laryngeal cancers. When 48 samples were studied, the expression level of miR-455-5p was significantly higher in cancers compared with adjacent noncancerous counterparts and benign laryngeal tissues (p = 0.0113). Furthermore, expression level of miR-196a was clearly higher in cancer tissues compared with noncancerous other tissues (adjacent noncancerous counterparts and benign laryngeal tissues, p = 0.0003; dysplasias, p = 0.0040) (Figure 3A). Although miR-133b showed significantly lower expression levels when cancer samples were compared with matched noncancerous laryngeal tissues (Figure 2B), expression level of this miRNA was not significantly lower in cancer samples when compared with noncancerous other tissues (noncancerous counterparts and benign laryngeal tissues, p = 0.8353; dysplasias, p = 0.2185) in the study using multiple samples (Figure 3B).

**Figure 4:**
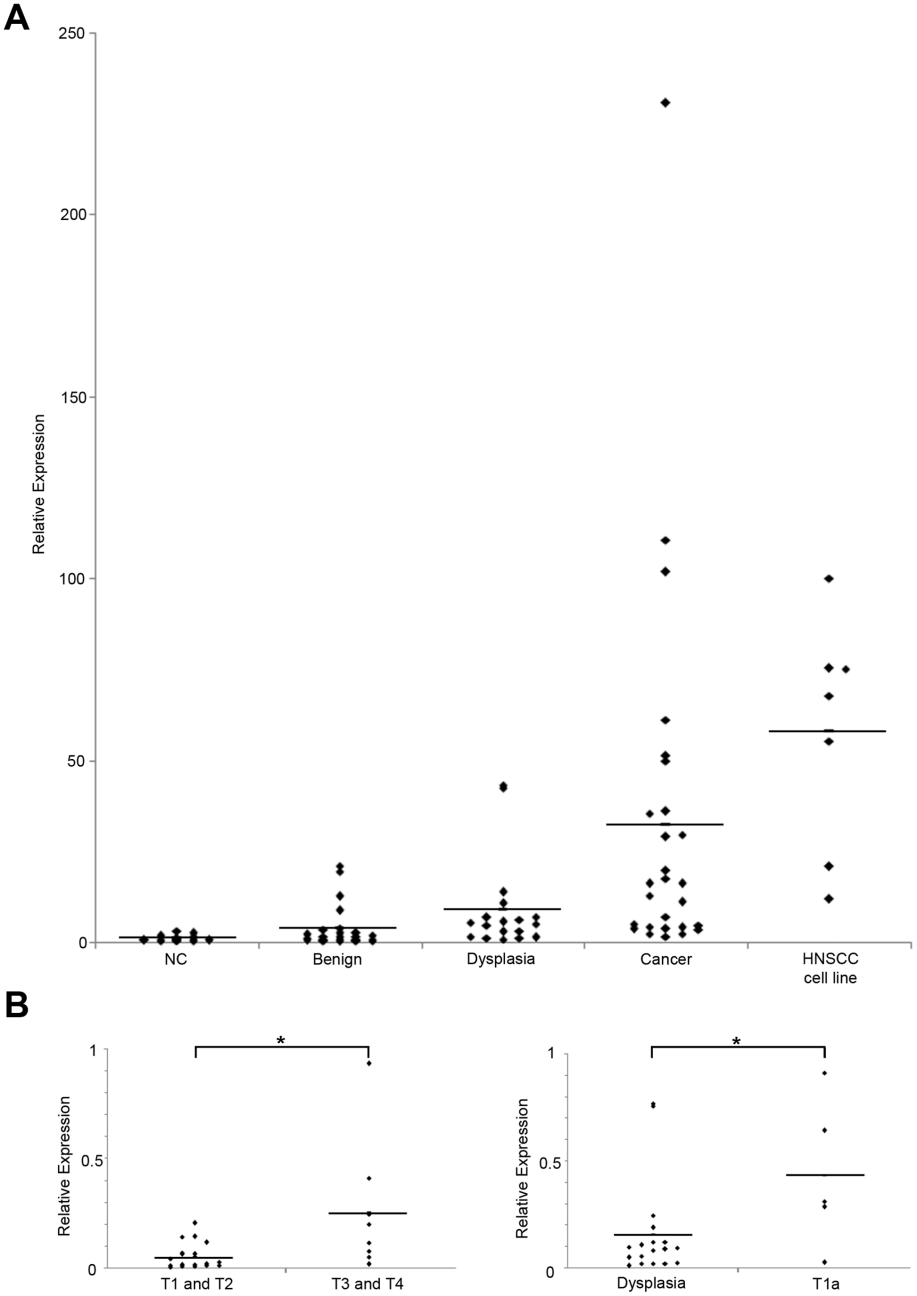
miR-196a as a potential diagnostic marker for laryngeal cancer. (A) The significance of miR-196a as a promising biomarker for laryngeal cancer was confirmed by TaqMan® qRT-PCR using 84 tissue samples. The assay also included 7 HNSCC cell lines. Noncancerous counterparts (NCs) are shown as open quadrangles. Bars indicate mean value of each group. (B) Expression level of miR-196a was higher in advanced (T3 and T4) cancers compared with early cancers (T1 and T2) (*left panel*). Furthermore, significantly higher level of miR-196a expression was observed in early T1a cancer samples compared with precancerous dysplasia samples (*right panel*). Bars indicate mean value of each group. *, *p*<0.05.

Therefore, to further explore the significance of miR-196a as a promising biomarker for laryngeal cancer, qRT-PCR analysis of miR-196a was performed on 84 histologically verified samples (15 noncancerous counterparts, 24 benign diseases, 18 dysplasias, 27 laryngeal cancers) and 7 HNSCC cell lines. The study showed that increasing tendency of miR-196a expression level was observed when cancer samples were compared with noncancerous counterparts, benign tissues, or dysplasias (Figure 4A). The expression of miR-196a in cancers was significantly higher than their matched paired samples (p = 0.005) and was also evident in laryngeal cancer cell line JHU-011 cells (data not shown). Furthermore, we found the expression of miR-196a was significantly higher in advanced cancer than early cancer (p = 0.0045; Figure 4B, *left panel*), and also in early cancer than precancerous dysplasia (p = 0.0263; Figure 4B, *Right panel*). Together with these findings, miR-196a could be a favorable marker for laryngeal cancer.

### Qualitative Difference of miR-196a isomiRs Revealed by Deep Sequencing

The recent advent of deep sequencing technology revealed that there are usually variations in mature miRNA sequences as coined with the term “isomiR” [45]. Such variations are primarily caused by a shift of cleavage sites when mature miRNAs are processed by Drosha and Dicer, but are also introduced by terminal nucleotide additions or internal RNA editing. Although physiological meanings for diversity of isomiR remain elusive, accumulating evidences suggest that there exist developmental/tissue-specific preference in the spectrum of isomiRs.

**Figure 5:**
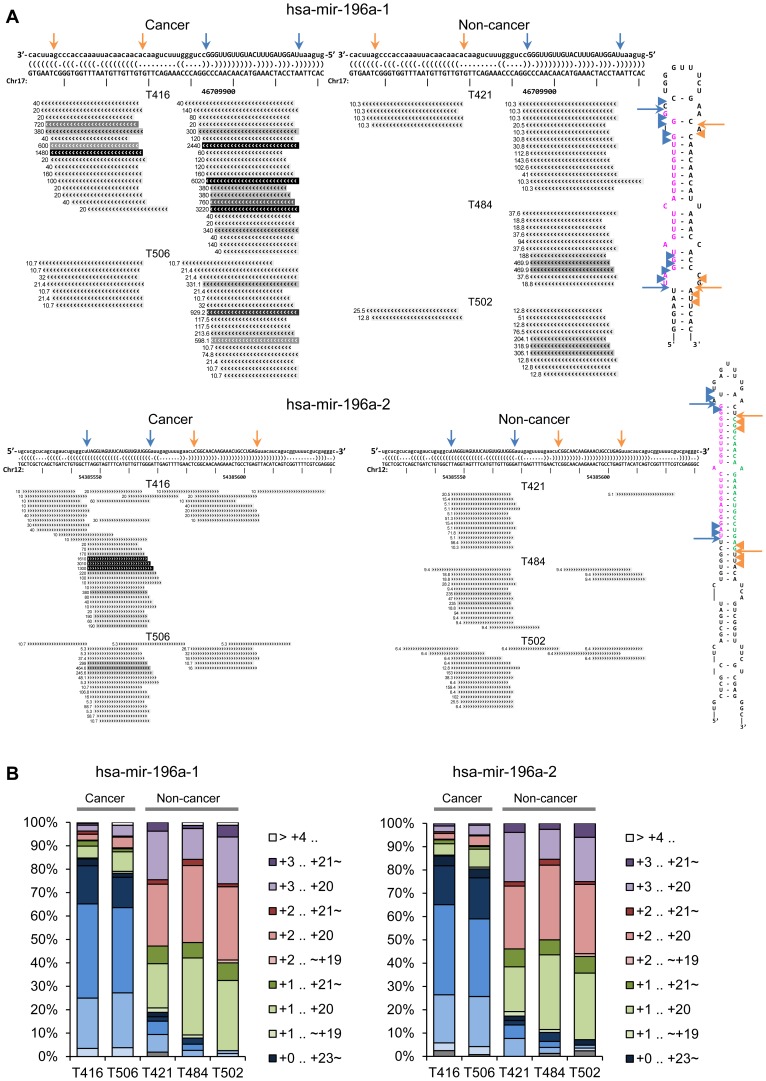
The repertoire of miR-196a isomiRs in laryngeal cancer and non-cancer tissues. (A) Expressions of miR-196a isomiRs were compared between 2 laryngeal cancer (T416 and T506; *left panels*) and 3 non-cancer (T421, T484 and T502; *right panels*) samples. Short read alignments are shown against both mir-196a-1 precursor at chr17∶46709852-46709921[-] (GRCh37.p5 coordinates) (*upper panels*) and mir-196a-2 precursor at chr12∶54385522-54385631[+] (*lower panels*). Potential hairpin secondary structures of the precursor sequences are shown as dot-bracket notation (*top*) or stem-loop arrangement (*right*). *Arrows* and *arrowheads* notations indicate typical cleavage sites employed in miRBase entry and atypical cleavage sites revealed by our deep sequencing analysis, respectively. (B) Percentage size distributions for miR-196a isomiRs found in short read alignment against mir-196a-1 precursor (*left*) and mir-196a-2 precursor (*right*). Two advanced T3 laryngeal cancer (T416 and T506) and 3 non-cancer laryngeal papilloma (T421, T484 and T502) samples are compared in parallel. Position designation for isomiRs (e. g. +0. +23) is in relative to the start site of the mature miR-196a miRBase entry as +0.

Thus, to explore the possible heterogeneity in the expression profiles of miR-196a isomiRs between laryngeal cancer and non-cancer tissues, deep sequencing analysis of small RNA was conducted by using Applied Biosystems SOLiD system (Kamimoto T et al., manuscript submitted). There are two distinct genes for miR-196a, namely *mir-196a-1* at chr17 and *mir-196a-2* at chr12, and their transcribed mature miRNAs share the same 5′ arm sequence (miR-196a-5p, referred shortly as miR-196a) with their 3′ arm sequences slightly different. At both *mir-196a-1* and *mir-196a-2* loci, the deep sequencing results showed that the normalized read counts (reads per million mapped reads; RPM) were significantly higher in cancer samples as compared with non-cancer samples (Figure 5A), consistent with the quantitative data of miR-196a obtained as above.

In addition to these quantitative results, we also found that a possible qualitative difference could be deduced from the deep sequencing results of cancer and non-cancer samples. Among a series of miR-196a isomiRs detected in each sample, the most abundant ones in cancer samples were nearly equal to the canonical sequence deposited in miRBase entry (MIMAT0000226, 5′- uagguaguuucauguuguuggg −3′), while those in non-cancer samples were shorter at both ends with 1–2 nucleotides (Figure 5B). Such a truncation of mature miRNA sequence was not necessarily observed with other miRNAs in our study, so it is not likely caused by non-specific degradation of deep sequencing samples. These data cumulatively suggest that expression of mature miR-196a is maintained low and with a truncated form in normal laryngeal tissues, and is up-regulated with a complete length in laryngeal cancers.

### miR-196a Detection and Localization in Laryngeal Cancer by *in situ* Hybridization

**Figure 6:**
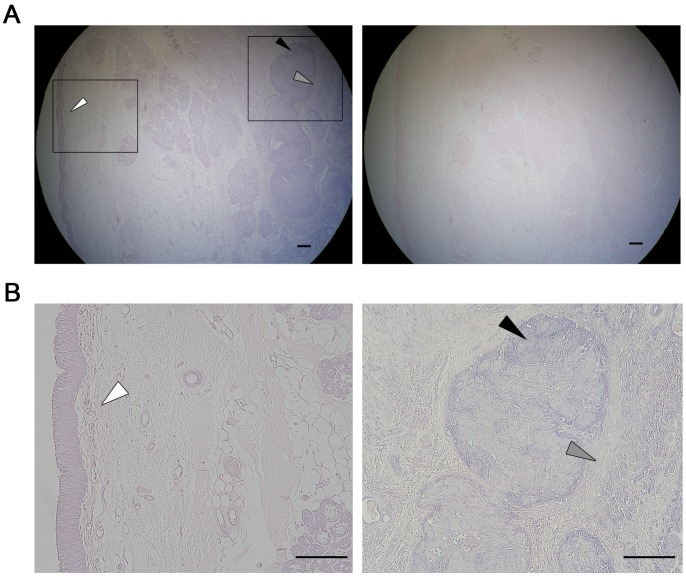
*In situ* localization of miR-196a in laryngeal tissues. (A) 3′-DIG–labeled LNA probe was utilized to detect the localization of miR-196a in laryngeal cancer tissues by *in situ* hybridization. Negative control without miR-196a-specific probe is shown (*right panel*). *Boxed fields* are presented with higher magnification in panel B. (B) miR-196a was detected both in cancer cells (*right panel*; *filled arrowheads*) and stroma (*right panel*; *grey arrowheads*), while not detected in normal squamous cell epithelium (*left panel*; *white arrowheads*). The scale bar (*black*) represents 200 µm.

To verify the expression of miR-196a in laryngeal cancer tissue, we conducted *in situ* hybridization for mature miR-196a on paraffin-embedded tissue slices of both advanced T3 laryngeal cancer and normal mucosa obtained from unaffected side of the same clinical specimen. We utilized Exiqon’s miRCURY DIG-labeled LNA detection probe which successfully detected the expression of miRNA in FFPE sample in a recent report [46]. The result showed that the expression of miR-196a was evident in laryngeal cancer, but was under detection levels in the adjacent normal counterpart (Figure 6). Interestingly, the expression of miR-196a was slightly detected in the stromal area next to cancer in the FFPE sample. Together with the result that miR-196a is highly expressed in laryngeal cancer cell line JHU-011 cells (Figure 4A), these findings raised the possibility that cancer cells secrete miR-196a transferable or functional in stromal cells.

### Effect of miR-196a Inhibition on Laryngeal Cancer Cell Viability

**Figure 7:**
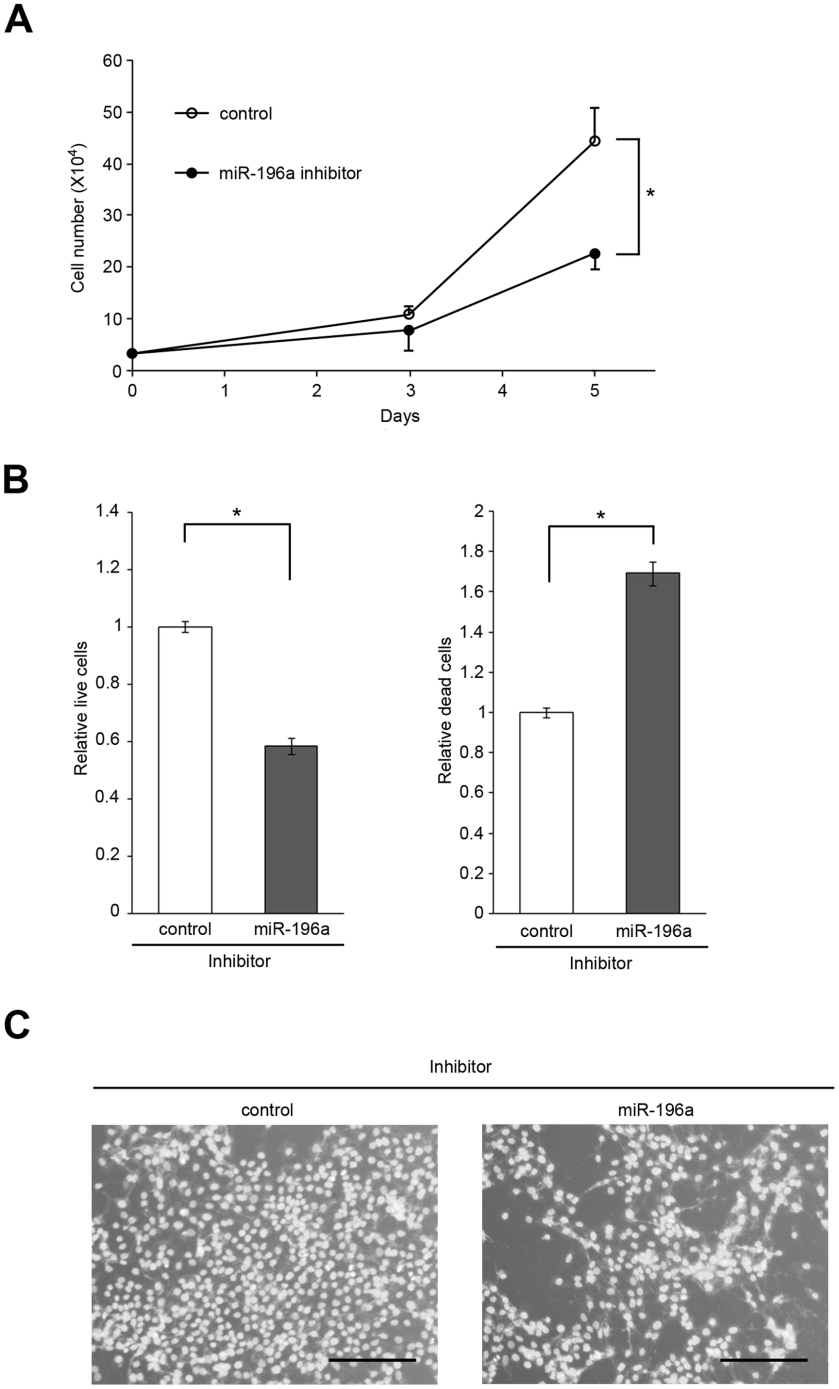
Reduced proliferation and viability of laryngeal cancer cells by miR-196a inhibition. (A) Laryngeal cancer-derived JHU-011 cells were transfected with either miR-196a inhibitor or Inhibitor Negative Control (control) on day 0 and cell numbers were counted up to 5 days to evaluate the effect of miR-196a inhibition on the cell proliferation. Significant suppression of cell proliferation was observed on day 5. Similar results were obtained in at least three independent experiments. *, *p*<0.05. (B) JHU-011 cells were transfected with either miR-196a inhibitor or Inhibitor Negative Control (control) to examine the effect of miR-196a inhibition on the cell viability. 72 hours after transfection, the numbers of both live cells and dead cells were assessed by using MultiTox-Fluor Multiplex Cytotoxicity Assay. Live cell fluorescence and dead cell fluorescence revealed significant suppression of relative live cell number (*left panel*), while miR-196a inhibition significantly increased relative dead cell number as compared to control (*right panel*). Data are expressed as mean values with standard deviations. Similar results were obtained in at least three independent experiments. *, *p*<0.05. (C) Cells transfected with either miR-196a inhibitor or control were cultured for 3 days followed by staining with Hoechst 33258. Representative pictures are shown. *, *p*<0.05. The scale bar (black) represents 200 µm.

Next, to explore the physiological significance of miR-196a expression in laryngeal cancer, we first examined whether miR-196a is beneficial to the propagation of laryngeal cancer cells. JHU-011 cells derived from clinical laryngeal cancer were transfected with either miR-196a inhibitor or control, and after culture for 5 days the cells were subjected to both cell growth and survival analysis in the presence of miR-196a inhibitor. Significant suppression of cell proliferation was observed by miR-196a inhibitor compared with control (miR-196a inhibitor, 22.67±3.06×10^4^/ml; control, 44.33±6.66×10^4^/ml; p = 0.0069) (Figure 7A).

Furthermore, cell viability and cytotoxicity were confirmed by the MultiTox-Fluor Multiplex Cytotoxicity Assay (Promega, Fitchburg, WI) at 3 days after transfection. This ratiometric fluorescent assay uses two substrates, GF-AFC that is cell permeable to assess the viable cells and bis-AAF-R110 that is not cell permeable to assess the protease activity from dead cells. Mean relative fluorescence unit of live cells or dead cells treated with control was set at 1 and the effect of miR-196a inhibition was evaluated. The study showed that relative number of live cells or that of dead cells were significantly suppressed (control, 1±0.019; miR-196a inhibitor, 0.584±0.029; p = 0.0022) or enhanced (control, 1±0.025; miR-196a inhibitor, 1.692±0.059; p = 0.0015), respectively, in the miR-196a inhibitor treatment group compared with control group (Figure 7B). Suppression of cell growth by miR-196a inhibitor was also visualized by Hoechst 33258, a nuclear stain that emits blue fluorescence when bound to double-stranded DNA (Figure 7C).

### Effect of miR-196a Inhibition on Tumor Growth and Metastasis of Laryngeal Cancer Xenografts

**Figure 8:**
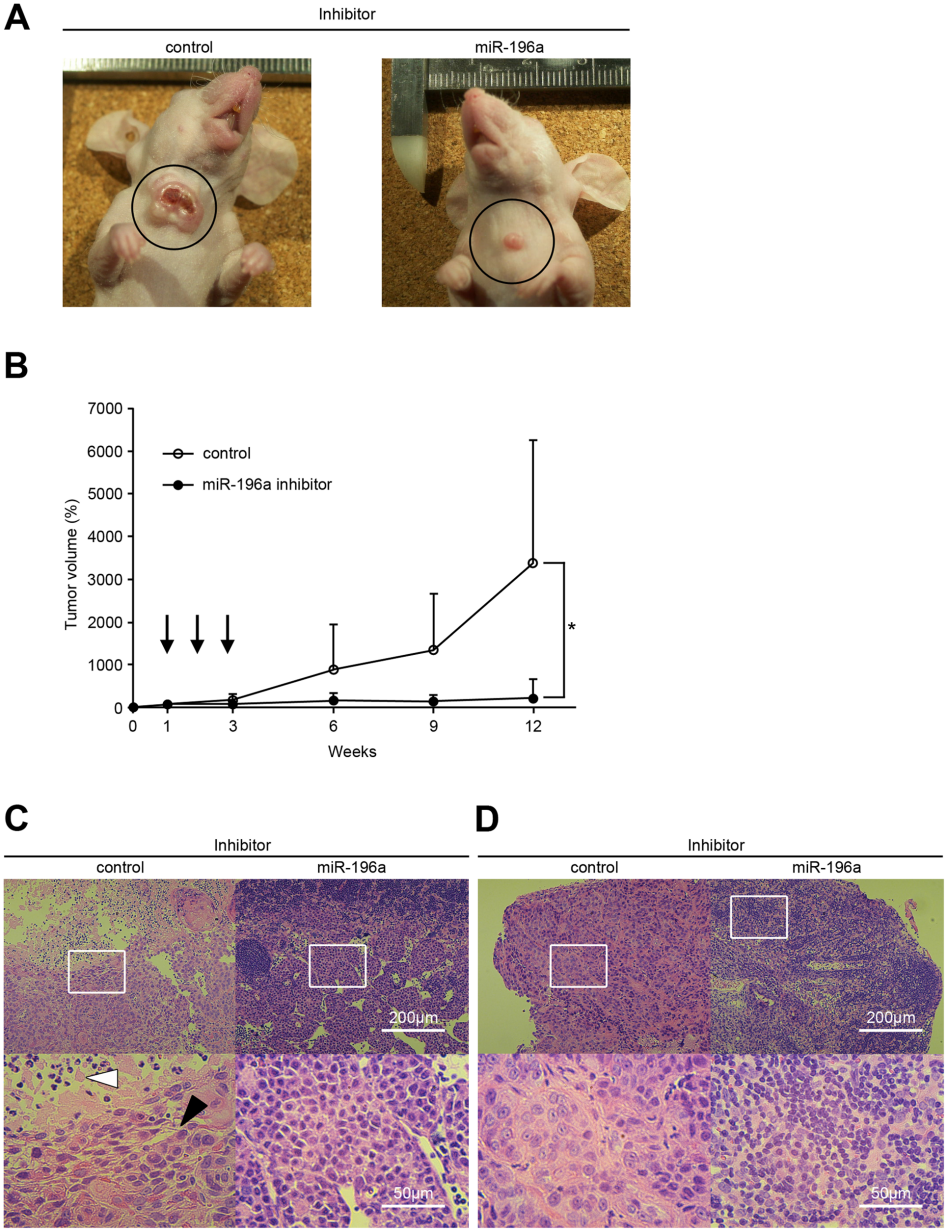
Head and neck cancer growth suppression in an orthotopic murine model by miR-196a inhibition. (A) Nude mice were injected with JHU-011 cells at the level of hyoid bone and then treated with miR-196a inhibitor or control. Representative mice were photographed on 9 weeks after inoculation of JHU-011 cells, showing the obvious treatment effect of miR-196a inhibitor. (B) Inhibitors were administered into the subcutaneous spaces around the tumors at the indicated time points of 7, 13, and 19 days after inoculation (arrows). The tumor volume at 7 days after inoculation was set as 100% and measured up to 12 weeks after surgery. Data are shown as mean values with standard deviations in each treatment group. Significant tumor growth suppression by miR-196a inhibitor was observed at 12 weeks after inoculation. *, *p*<0.05. (C) Histological analysis of primary tumor at 12 weeks after inoculation. While squamous cell cancer cells (white arrowhead) with central necrosis (filled arrowhead) were observed in control (*left panels*), tumor cells were replaced with histiocytes after miR-196a inhibition (*right panels*). The scale bar (white) represents 200 µm or 50 µm, as shown in *right panels*. (D) Histological analysis of locoregional lymph nodes at 12 weeks after inoculation. Representative pictures of control group with metastatic tumor cells (*left panels*) together with the pictures of miR-196a inhibitor group without tumor cells (*right panels*) are shown. The scale bar (white) represents 200 µm or 50 µm, as shown in *right panels*. (C, D) High magnification images of boxed fields in upper panels are shown in lower panels and FFPE sections were stained with hematoxylin and eosin.

The results demonstrated above clearly indicate that miR-196a is up-regulated in laryngeal cancer cells to support their proliferation. Therefore, to examine whether inhibition of miR-196a expression could counteract the propagation of laryngeal cancer *in* *vivo*, orthotopic xenograft model in mice was employed. The volume of each tumor at 7 days after inoculation was set as 100% and measured up to 12 weeks after injection of JHU-011 cells. Significant suppression of the tumor volume was observed at 12 weeks after inoculation in the miR-196a inhibitor treatment group compared with control (control, 3397±2885%; miR-196a inhibitor, 226±476%; p = 0.0415) (Figures 8A and B).

Histological analyses revealed the growth of squamous cell cancer with central necrosis cells in the control treatment group. On the other hand, cancer cells were replaced with histiocytes with no residual cancer cells in the miR-196a inhibitor treatment group (Figure 8C). Furthermore, suppression of locoregional metastasis was observed in the miR-196a treatment group while clear metastatic cancer cells were observed in the control (Figure 8D).

## Discussion

Previous work has shown that specific miRNA expression signatures in various human cancers are potentially used to diagnose the tumor types, to estimate the prognosis, or to predict the response to chemotherapy [15]. Each miRNA is considered to control the expression of hundreds of mRNA species to regulate a whole network of interacting molecules [47]. miRNAs also display extraordinary tissue-specificity, and their tissue-specific nature has already been exploited for the purpose of diagnosing and classifying primary cancers and their metastases [17], [48]. Recent reports revealed that manipulation of tumor-specific miRNAs could suppress cell invasion[23]–[26], cell migration [24], and to reduce the anchorage-independent growth [26] in vitro, and suppress metastasis in vivo [25]. Several known mechanisms to explain the tumor suppressive role by miRNAs modifications are epigenetic aberration by targeting the DNA methyltransferases [23], regulation of the epidermal growth factor receptor expression [24], and regulation of tumor angiogenesis [26].

Moreover, it has been suggested that specific miRNAs may have crucial roles in the initiation and/or progression of human cancers through their effects on various molecular pathways. A better understanding of miRNA expression in cancer may uncover novel molecular pathways, or novel mechanisms of activation for known pathways [19], [22], [23], [25], [49], [50]. We have obtained miRNA expression signatures in laryngeal cancer and identified several miRNAs differentially expressed in laryngeal cancer tissues as potential biomarkers and therapeutic targets for laryngeal cancer. Compared with previous studies in HNSCC, we found similar trend of deregulation in miR-133b [51], while 3 other miRNAs found to be deregulated in laryngeal cancer were not reported before. There are several possible explanations for this discrepancy of the findings between previous reports and ours. First, the array platform used for the identification of miRNA is different and may yield different patterns. Second, the material we utilized as normal control is different than what was used in previous studies and the choice of normal is known to influence the outcome of gene profiling analysis [52] [53].

### miRNAs Down-regulated in Laryngeal Cancer

miRNAs are often located in genomic unstable regions in cancers and therefore are typically down-regulated in tumors and have the potential to act as tumor suppressors [15], [54]. Down-regulation of miRNAs in cancers may be achieved through mutation or by epigenetic silencing or the miRNA, resulting in loss of tissue-specific miRNA synthesis and overexpression of oncogenes which normally function as tumor suppressors [55]. Down-regulation of miR-133b was reported in colorectal [36], bladder [34], and tongue [51] cancer. The potential target of the miR-133b has been reported to be oncogenic KRAS [36].

In the previous reports, miR-145 was reported as miRNA down-regulated in multiple cancers as mentioned above[32]–[43]. Recent report using microarray and qRT-PCR showed down-regulation of miR-145 in laryngeal cancer consistent with our result [56]. MYCN, FOS, YES, FLY, cyclins D2 and L1, MAP3K3 and MAPK4K4 were reported to be potential oncogenic targets of miR-145 [33]. miR-145 is related to angiogenesis and down-regulated in the lungs when exposed to cigarette smoke [57]. Furthermore, the proto-oncogene YES1 and the transduction protein MAP3K3 were reported to be common potential targets of miR-133b and miR-145 [36].

### miRNAs Up-regulated in Laryngeal Cancer

Although miRNAs are often down-regulated in cancers, miRNAs can also be up-regulated in cancers and act as oncogenes [13], [54]. While miR-130b-5p, miR-455-3p, miR-455-5p, and miR-801 were up-regulated in laryngeal cancer in our study, much less study has been performed on these miRNAs before. miR-130b-3p complementary to miR-130b-5p was reported to be related to schizophrenia by targeting MECP2 protein [58]. miR-455-3p has been reported to be differentially expressed in smokers compared to nonsmokers [59]. One known target of miR-455-3p is TTK (also known as MPS1) protein kinase [59] which regulates mitotic spindle formation and cell proliferation [60] [61]. TTK kinase is differentially expressed in lung, esophageal [62] and breast [63] cancer, and mutation of TTK kinase induces the failure of the spindle checkpoint and aberrant mitosis of cells.

Interestingly, recent study have reported that decreased expression of miR-455-5p was correlated with vascular invasion, poor overall survival, and poor disease-free survival in endometrial serous adenocarcinoma suggesting the potential involvement of miR-455-5p in cancer progression [44]. Up-regulated miR-455-5p may have different roles in the biology of laryngeal cancer compared with endometrial carcinoma.

### miR-196a as the Most Advantageous Biomarker Up-regulated in Laryngeal Cancer

miR-196a showed most dramatic expression pattern in laryngeal cancer compared with other miRNAs differentially expressed in laryngeal cancer. Notably, compared with pre-cancerous dysplasias, miR-196a expression was significantly higher in early T1a cancer in our study, suggesting the potential of miR-196a to be a very supportive or crucial tumor marker especially for early laryngeal cancer with frequent difficulty with pathological diagnosis [64]. miR-196a is located in HOX gene clusters [65] and this microRNA has been suggested to potentially target HOXB8, HOXC8, HOXD8 and HOXA7 [66]. HOX proteins are major transcription factors that play a crucial role during embryogenesis, organogenesis and oncogenesis [67]. Another previous report proved that miR-196a promoted cell proliferation, anchorage-independent growth and suppressed apoptosis by targeting annexin A1 [68]. Furthermore, high levels of miR-196a activates the AKT signaling pathway and promotes cancer cell detachment, migration, invasion, but does not impact on proliferation or apoptosis in the colorectal cancer cell line [69]. Recently, it has been reported that miR-196a has a role in differentiation and proliferation of human adipose tissue-derived mesenchymal stem cells [70]. Furthermore, the role of miR-196a as one of the regulators of the ETS transcription factor ERG known as an adverse prognostic factor for acute leukemia has been also reported [71]. Deregulated expression of HOX genes including HOXB8, HOXC8, HOXD8 and HOXA7 in esophageal squamous cell carcinoma have been reported [72], [73], and miR-196a is significantly up-regulated in pancreatic [74], breast [75], and esophageal [68] cancer. Although further study is necessary to examine biological significance including the downstream targets of miR-196a in laryngeal cancer, there may be a mechanism through HOX gene deregulation to promote laryngeal cancer.

### Heterogeneity of miR-196a isomiRs in Deep Sequencing Data

By employing deep sequencing technique, the multiplicity and other frequent repertoire of isomiRs could be detected with miR-196a, including those are not annotated in the current release of miRBase. There are 2 genomic loci that produce the same mature miR-196a-5p sequence, namely mir-196a-1 in chromosome 17 and mir-196a-2 in chromosome 12. In the current release of miRBase, both miR-196a-5p and -3p arm sequences are annotated in mir-196a-2 precursor, while miR-196a-5p but not -3p sequence is annotated in mir-196a-1 precursor. According to our deep sequencing data, mir-196a-1 precursor is as well responsible for expressing unannotated miR-196a-3p sequence that is slightly different from that of mir-196a-2 precursor origin. It has been suggested that there are tissue-dependent preference of isomiR [76], and our data revealed that miR-196a isomiRs are differentially expressed between cancer and non-cancer samples in terms of both quantitative and qualitative manner. In cancer tissues, the expression level of miR-196a isomiRs is upregulated and the length spectrum of miR-196a isomiRs is peaked at those found at miRBase entry of miR-196a. In contrast, non-cancer tissues revealed low expression level of miR-196a in a form shorter than miRBase information. Although the mechanism underlying this is not clear, such quantitative and qualitative traits of miR-196a characteristic to laryngeal cancer will help their use as a biomarker.

### Stable Detection of miR-196a Expression in Surgical Laryngeal Cancer Specimen

A relevant feature of miRNA biology is their remarkable stability to enable the extraction and evaluation of disease-specific miRNAs in tissue samples even after FFPE processing [18]. Furthermore, detection of miRNAs in FFPE samples could provide valuable information on the cellular localization of relevant miRNAs in disease processes [77]. In our study, miR-196a was detected both in cancer cells and cancer-related stroma as miR-21 expression in esophageal squamous cell carcinoma [78]. As mentioned in cardiac miRNAs in a recent report [79],miR-196a could be produced by stromal cells with a paracrine function. Furthermore, laryngeal cancer tumor cells may secrete tumor-derived exosomes containing miRNAs [80] into the surrounding tissue which could be functional [81] in the stroma to promote tumorigenesis through several mechanisms including tumor angiogenesis [82]. Although the underlying biological role is still obscure, tumor-derived exosomes are definitely secreted into vascular flow carrying a subgroup of miRNAs as their contents. Actually, our preliminary data revealed that miR-196a could be detected in a serum sample obtained from advanced cancer patients (data not shown). Our findings suggested that miR-196a could be a potential tumor marker in the FFPE tissue sample of laryngeal cancer. Future studies on the detection of miR-196a on a higher number of patients are warranted.

### Tumor Growth Suppression by miR-196a Inhibitor

To determine whether miR-196a mediates tumorigenesis in laryngeal cancer, we first evaluated the miR-196a inhibition in JHU-011 cell line primary derived from laryngeal cancer. In vitro study revealed the significant growth suppression of JHU-011 cells by miR-196a inhibitor. Subsequently, we injected miR-196a inhibitor into pre-established JHU-011 xenografts. Our approach closely mimicked a “treatment”, as it was performed in already grown tumors, where cells had already formed their network of contacts within the host body [83]. We believe that our positive data in this settlement are the most significant and interesting, as they show that miR-196a inhibition can reduce the growth of pre-established laryngeal cancer xenografts up to 12 weeks. Thus, our laryngeal cancer xenograft data demonstrate, as a whole, that miR-196a is sufficient to strongly enhance laryngeal cancer growth (even if we are not providing the precise biological mechanisms behind this dramatic effect) and, consequently, that the inhibition of miR-196a is necessary, and in fact effective, to reduce the in vivo growth of this tumor.

Altogether, our results represent the first step toward a possible use of miR-196a as molecular marker for laryngeal cancer, and set the base for the future clinical employment of miR-196a inhibitor for the suppression of laryngeal cancer growth.
